# Moderate Traumatic Brain Injury Causes Acute Dendritic and Synaptic Degeneration in the Hippocampal Dentate Gyrus

**DOI:** 10.1371/journal.pone.0024566

**Published:** 2011-09-13

**Authors:** Xiang Gao, Ping Deng, Zao C. Xu, Jinhui Chen

**Affiliations:** 1 Spinal Cord and Brain Injury Research Group, Department of Neurosurgery, Stark Neuroscience Research Institute, Indianapolis, Indiana, United States of America; 2 Department of Anatomy and Cell Biology, Indiana University School of Medicine, Indianapolis, Indiana, United States of America; University of North Dakota, United States of America

## Abstract

Hippocampal injury-associated learning and memory deficits are frequent hallmarks of brain trauma and are the most enduring and devastating consequences following traumatic brain injury (TBI). Several reports, including our recent paper, showed that TBI brought on by a moderate level of controlled cortical impact (CCI) induces immature newborn neuron death in the hippocampal dentate gyrus. In contrast, the majority of mature neurons are spared. Less research has been focused on these spared neurons, which may also be injured or compromised by TBI. Here we examined the dendrite morphologies, dendritic spines, and synaptic structures using a genetic approach in combination with immunohistochemistry and Golgi staining. We found that although most of the mature granular neurons were spared following TBI at a moderate level of impact, they exhibited dramatic dendritic beading and fragmentation, decreased number of dendritic branches, and a lower density of dendritic spines, particularly the mushroom-shaped mature spines. Further studies showed that the density of synapses in the molecular layer of the hippocampal dentate gyrus was significantly reduced. The electrophysiological activity of neurons was impaired as well. These results indicate that TBI not only induces cell death in immature granular neurons, it also causes significant dendritic and synaptic degeneration in pathohistology. TBI also impairs the function of the spared mature granular neurons in the hippocampal dentate gyrus. These observations point to a potential anatomic substrate to explain, in part, the development of posttraumatic memory deficits. They also indicate that dendritic damage in the hippocampal dentate gyrus may serve as a therapeutic target following TBI.

## Introduction

Traumatic brain injury (TBI) not only results in immediate CNS tissue disruption (primary injury), but also causes secondary damage among the surviving cells via complex mechanisms triggered by the primary event [1], [2]. This secondary injury initiated by the primary impact leads to persistent cognitive, sensory, and motor dysfunction [3]. The hippocampus undergoes similar neuropathological changes after both human closed-head injury and experimental injury models of TBI, including controlled cortical impact (CCI) injury [2], [4], fluid percussion [5], and stretch injury [6]. These common changes suggest that the hippocampus is particularly vulnerable to secondary injury following TBI [7]. The pathologies in the hippocampus play a leading role in the disturbance of learning, memory [8], and higher cognitive function [8]. Hippocampal injury-associated learning and memory deficits are frequent hallmarks of brain trauma and are the most enduring and devastating consequences following TBI [9]. Currently, there is no effective treatment that can preserve or restore such functions. Therefore, it is critically important to understand the molecular and cellular mechanisms behind learning and memory impairment following TBI in hopes of finding a way to prevent the progression of damage or encourage the recovery of learning and memory.

Although TBI induces cell death in the hippocampus, most of the neurons are spared following moderate or mild TBI. Less attention has been given to determine whether these spared neurons suffer injury. Injured animals also demonstrated a diffusive axonal injury [10], [11], [12] and profound loss of synapses in CA1 [13] with a significant deafferentation in electroactivity [14]. Less is known about potential indirect effects of TBI on dendrites. Light microscopic studies of MAP2 revealed a prominent loss of MAP2 immunofluorescence in apical dendrites of pyramidal neurons in the cortex [15], [16]. This suggests dendritic damage in the spared neurons after TBI.

In this study, we assessed the dendrites, dendritic spines, synapses, and function of spared granular neurons in the hippocampal dentate gyrus (HDG). The results showed extensively reduced dendritic branches in the spared granular neurons. The remaining dendrites exhibited swelling with beading, which is a hallmark of dendritic injury [17], [18], [19]. The density of dendritic spines and number of synapses were decreased, and electrophysiological activities were impaired. These data suggest, in addition to cell death and diffused axonal injury, extensive dendritic damage in the spared neurons could also disrupt neurocircuits and likely significantly contribute to functional impairment following TBI.

## Materials and Methods

### Animals

Mice were housed with a 12/12 light/dark cycle and had free access to food and water ad libitum according to the principles outlined in “Guidelines for Care and Use of Experimental Animals”. They were used in experiments at an age of 8–10 weeks. All procedures were approved by Indiana University IACUC. The approval number is 3332.

### Genetically labeled the granular neurons with Enhanced Green Fluorescent Protein (EGFP) in the HDG

We took advantage of a line of transgenic mice generated by Dr. Lowell and characterized by our laboratory [20], [21]. These mice allowed us to easily visualize and quantify dendrites of granular neurons in the HDG in high resolution. In this line of transgenic mice, a recombinase Cre, driven by the pro-opiomalanocortin (POMC) promoter, was expressed in the granular neurons of the HDG [20]. Based on the *Cre/loxP* conditional recombinase system [22], we crossed the POMC-Cre mice with Z/EG reporter mice [23] [Tg(ACTB-Bgeo/GFP021Lbe Stock#003920] to obtain POMC-Cre;EGFP double transgenic mice [21]. In these mice, EGFP is expressed after Cre-mediated deletion of loxP-flanked stop codon [23]. The granular neurons with Cre expression will simultaneously express EGFP. Through these genetic manipulations, we ectopically expressed EGFP in the HDG granular neurons. Immunostaining with EGFP antibody allowed us to easily visualize dentate gyrus granular neurons with their dendritic structures at a resolution high enough to image and quantify the number of dendrites and axons correctly projecting toward CA3.

The POMC-Cre mouse line (B6.129) was kindly provided by Dr. Joel K. Elmquist and Dr. Bradford B. Lowell at Beth Israel Deaconess Medical Center. Z/EG reporter mice were purchased from The Jackson laboratory. Two transgenic mouse lines with different genetic backgrounds were backcrossed to the C57 BL/6 line for more than 10 generations. Animals were genotyped by PCR using genomic DNA extracted from mouse tails using a Kit from Qiagen. Primer sequences are as follows: 5′-GAGATATCTTTAACCCTGATC -3′, 5′- TGGCTCAATGTCCTTCCTGG -3′ and 5′- CACATAAGCTGCATCGTTAAG -3′ for POMC-Cre. 5′- CTA GGC CAC AGA ATT GAA AGA TCT-3′, 5′- GTA GGT GGA AAT TCT AGC ATC ATC C -3′, 5′- AAG TTC ATC TGC ACC ACC G -3′ and 5′- TCC TTG AAG AAG ATG GTG CG -3′ for Z/EG reporter.

### Controlled Cortical Impact Traumatic Brain Injury

Male mice at 8–10 weeks old were subjected to moderate controlled cortical impact injury or sham treatment, as we previously described [24], [25], [26], with the following exceptions: the amount of deformation was set at 1.0 mm and the piston velocity controlled at 3.0 m/sec. These modifications result in a moderate level of injury using an electromagnetic model [27] (Impact One™ Stereotaxic Impactor for CCI, Leica Microsystem, Illinois USA). Briefly, the mice were anesthetized with avertin and placed in a stereotaxic frame (Kopf Instruments, Tujunga, CA) prior to TBI. Using sterile procedures, the skin was retracted and a 4 mm craniotomy centered between the lambda and bregma sutures was performed. A point was identified midway between the lambda and bregma sutures and midway between the central suture and the temporalis muscle laterally. The skullcap was carefully removed without disruption of the underlying dura. Prior to the injury, the impacting piston was angled so that the impacting tip (3 mm in diameter) was perpendicular to the exposed cortical surface. This was accomplished by rotating the entire stereotaxic frame on the transverse plane. The mouse CCI model uses an electromagnetic model [27] with which the experimenter can independently control the contact velocity and the level of cortical deformation to alter the severity of the injury. In these experiments, the contact velocity was set at 3.0 m/sec and the amount of deformation was set at 1.0 mm. These settings resulted in an injury of moderate severity. Sham (non-injured) animals received the craniotomy, but no CCI injury.

### Tissue Processing for Histology and Immunohistochemistry

At 72 hours after inflicting TBI, animals were deeply anesthetized with Avertin. They were then perfused transcardially with 0.9% saline, followed by a cold fixative containing 4% paraformaldehyde (PFA) in PBS. The brains were removed, post-fixed in 4% PFA overnight, and then cryoprotected with 30% sucrose for 48 hours. Serial coronal sections (30 µm thick) were cut using a cryostat (Leica CM 1950) and stored at −20°C. The sections were then processed for H.E staining, Nissl staining, or immunohistochemical analysis. Three sections from each injured mouse were selected for analysis. One section was at the epicenter (bregma −2.1 mm) while the other two were either 180 µm rostral or caudual to the epicenter. Three sections containing the hippocampus at the corresponding positions were selected from the sham treated mice as control.

### Hematoxylin and Eosin (H.E.) Staining

The brain sections were stained in hematoxylin for 6 minutes, rinsed with ddH_2_O, then decolorized in acid alcohol for 1 second. The sections were then rinsed with ddH_2_O before being immersed in Lithium Carbonate for 3 seconds and counterstained in Eosin for 15 seconds. Thereafter the sections were rinsed with ddH_2_O and dehydrated with 95% EtOH for 2 to 3 minutes and 100% EtOH for 2 to 3 minutes. They were then cleared with xylene for 2 to 5 minutes. The sections were finally mounted with DPX in a fume hood.

### Nissl Staining

The brain sections were stained in 0.1% cresyl violet solution for 5–10 minutes and then rinsed quickly in distilled water. After differentiating in 95% ethyl alcohol for 10 minutes, the sections were dehydrated in 100% alcohol for 2 to 5 min and then cleared in xylene for 2 to 5 min. The sections were then mounted with DPX.

### Immunohistochemistry

Immunofluorescent staining was carried out as follows: sections were rinsed in PBS 3 times and incubated in blocking solution (0.1% Triton X-100, 1% bovine serum albumin, and 5% normal goat serum in PBS) for 1 hour at room temperature. This was followed by an overnight incubation with primary antibody at 4°C. Sections were then washed and incubated with the secondary antibody for 2 hours at room temperature. After being treated with 4′, 6-Diamidino-2-phenylindole (DAPI) for 2 minutes, sections were washed with PBS 3 times and mounted using Fluorescentmount G (Sigma). Primary antibodies and their final concentrations were as follows: GFP antibody (1∶1000, rabbit, Invitrogen), and anti-synaptophysin antibody (1∶1000, mouse, Millipore). Secondary antibodies from Jackson ImmunoResearch Laboratories Inc. were all applied at the same dilution of 1∶1000. The images were taken at a primary magnification of 63× using an invert microscopy system (Zeiss, Axiovert 200 M) interfaced with a digital camera (Zeiss, Axio Cam MRc5) controlled by a computer.

### Assessing cell density in the hippocampal dentate gyrus

Stereological analysis of cell density in the HDG was performed as we previous described with modification [28]. Briefly, an investigator blind to the animal surgery performed quantitative stereologic analysis of cell number and layer volume in the dentate gyrus using DAPI stain to identify all cells. Three sections from each brain after TBI (n = 4) and each brain after sham-treatment (n = 4) were selected and processed for DAPI staining. These sections included one at the epicenter, one at 240 µm rostral to epicenter, and one 240 µm caudal to the epicenter. Total cell number was estimated using the optical fractionator method [29] and Bioquant software (Dan Diego, CA). Using accepted anatomic boundaries (The Mouse Brain, 2003 Paxinos and Franklin), the relevant structure was traced at low power (4×). The live image was generated with a Olympus BK51 microscope and a digital video camera. Pilot studies determined the dissector dimensions. We counted approximately one cell per sampling frame and allowed for a guard zone above and below the sampling site. A 40×objective was used to achieve optimal optical sectioning during stereologic analysis. The Stereo Investigator software placed dissector frames using a systematic-random sampling design within each contour. Only DAPI-positive ‘caps’ were counted, Cells defined as DAPI-positive that came into focus while focusing down through the dissector height. Dentate granule cell layer volume estimates were calculated using the Cavalieri principle and contours traced at low power.

### Golgi Staining

We used the FD Rapid Golgi Stain kit (FD NeuroTechnologies) to perform Golgi staining following the vendor's protocol. Briefly, the freshly dissected brains were immersed in impregnation solution (made by mixing equal volumes of Solutions A and B) and stored at room temperature for 2 weeks in the dark. The brains were then transferred into solution C and kept for 48 hrs at 4°C in the dark. Afterward, they were sliced using a horizon sliding slicer (SM2010R; Leica, Nussloch, Germany) at a thickness of 150 µm and stained using standard staining procedures.

### Quantification of Dendritic Injury

Disconnected dendritic beads (bulbs) and swellings are hallmarks of dendritic injury. In order to analyze the dendritic morphologies of EGFP-positive neurons, ten high magnification (63×) images were captured randomly in the molecular layer region close to the pial surface for each epicenter section. Then the images were taken at a primary magnification of 63× using an invert microscopy system (Zeiss, Axiovert 200 M) interfaced with a digital camera (Zeiss, Axio Cam MRc5) controlled by a computer. The number of immunopositive bulbs was manually counted. The evaluation of dendrite injury as well dendrite morphology and spine quantification at the following were done by a blinded analyzer. The dendrite beading density from the same animal was averaged and expressed as beading number/mm^2^.

### Dendrite Morphology Analysis

Granule neurons in the epicenter were analyzed using the following selection criteria: the neurons had to be fully impregnated and located in GCL with dendrites extending toward the molecular layer pial surface without truncated branches. For each selected neuron, all branches of the dendritic tree were reconstructed at 40× magnification using a motorized microscope (Olympus BX60) with Neurolucida software (Microbrightfield, VT). A 3D analysis of the reconstructed neurons was performed using NeuroExplorer software (Microbrightfield). Ten neurons were studied for each of the 5 animals in different experimental groups. Neurons from the same animal were averaged. Several aspects of dendritic morphology were examined. To assess overall changes, total length of dendrite trees and number of dendritic branches were compared across groups using Student T-test. The complexity of dendritic trees was assessed with the Sholl analysis [30], [31]. The number of intersections of dendrites was calculated with concentric spheres positioned at radial intervals of 10 µm.

### Spine Quantification

For analysis of dendritic spine morphology, high magnification images were captured using a camera (DXC-390, Sony Corporation, Tokyo, Japan) attached to an Olympus upright microscope (BX60, Japanese). These images were magnified with the100× oil immersion objective for each section from control mice or injured mice. Each image contained a *Z*-stack maximum projection of a dendrite from a DG granular cell (25 dendritic segments with >1500 spines for each mouse were counted). To minimize bias, only spines that emerged perpendicular to the dendritic shaft were counted. To assess the TBI-induced changes in spine morphology, spines in the selected segments were classified into mushroom, stubby, and filiopodia categories. The proportion of spines in each category was compared using Student T-test.

### Synaptic Quantification

Synaptic densities were analyzed with Image J. Ten highly magnified images were captured randomly in the molecular layer for each epicenter section. Then the threshold was determined to outline the synaptophysin immunopositive puncta, and the number of puncta was detected using the “analyze particle” module of the program. The synaptic densities from the same animal were averaged and normalized with data from the control group to determine the relative synaptic density.

### Electrophysiology

To prepare brain slices, mice were anesthetized with ketamine-HCl (100 mg/kg, i.p.) and the brain was quickly removed 3 days after surgery. Transverse hippocampal slices (400 µm) were cut using a vibratome (VT1000S; Leica, Nussloch, Germany) in an ice-cold (4°C) sucrose solution containing (in mM): 230 sucrose, 26 NaHCO_3_, 2.5 KCl, 1.25 NaH_2_PO_4_, 0.5 CaCl_2_, 10 MgSO_4_, 10 glucose, pH 7.4, 290–305 mOsm/L, equilibrated with 95% O_2_ and 5% CO_2_. The slices were maintained in an artificial cerebrospinal fluid (ACSF) containing (in mM): 130 NaCl, 3 KCl, 2 CaCl_2_, 2 MgCl_2_, 1.25 NaH_2_PO_4_, 26 NaHCO_3_, and 10 glucose, pH 7.4, 295–305 mOsm/L. The ACSF was continuously equilibrated with 95% O_2_ and 5% CO_2_, and slices were incubated for >1 h before recording. Recording electrodes were prepared from borosilicate glass (World Precision Instruments, Sarasota, FL) using a horizontal electrode puller (P-97; Sutter Instruments, Novato, CA). Electrodes had resistances of 3–5 MΩ when filled with an intracellular solution. Oxygenated ACSF was used as a bath solution, and the flow rate was adjusted to 2–3 ml/min. Granule cells in the dentate gyrus (ipsilateral to the trauma) were visualized with an infrared-differential interference contrast (DIC) microscope (BX50WI; Olympus Optical, Tokyo, Japan) and a CCD camera. All recordings were performed at 24°C with an Axopatch 200B amplifier (Molecular Devices, Foster City, CA).

During whole-cell recordings, series resistance (8–20 MΩ) was monitored periodically, and cells with >15% change were excluded from the analysis. Signals were filtered at 1 kHz and digitized at a sampling rate of 5 kHz using a data-acquisition program (Axograph 4.6; Molecular Devices). For whole-cell current-clamp recording, electrodes were filled with an intracellular solution containing (in mM): 125 KMeSO_4_, 20 KCl, 1 MgCl_2_, 1 EGTA, 0.2 CaCl_2_, 10 HEPES, 2 Mg-ATP, and 0.4 Na-GTP (pH 7.4, 280–295 mOsm/l). The fast *I*-clamp mode was used. Depolarizing current pulses (600 ms, 20–200 pA) were applied to evoke either a single firing or repetitive firings. To record miniature excitatory postsynaptic currents (mEPSCs), electrodes were filled with an intracellular solution containing (in mM): 92 CsMeSO_4_, 43 CsCl, 1 MgCl_2_, 2 EGTA, 5 TEA, 10 HEPES and 2 Mg-ATP, pH 7.4, 295–300 mOsm/L. Granule cells were clamped at −70 mV. In addition, tetrodotoxin (1 µM) and bicuculline (30 µM) were applied in the bath solution.

### Statistical Analysis

The collected data were expressed as mean ± standard deviation and the differences were analyzed using Student's t-test with the significant level set at *P*<0.05.

## Results

### The anatomical structure of the hippocampus remains intact following moderate TBI

C57/BL6 mice at the age of 8–10 weeks old were subjected to moderate CCI-injury (n = 5) or sham treatment (n = 5). Seventy-two hours after surgery, the mice were perfused and their brains were removed in order to assess the architectures of the hippocampi with H.E. staining and Nissl staining. At the time point, the spared neurons in the HDG showed the peak of dendrite damage and synaptic degeneration (unpublished data). Differentiated neurons in the hippocampus proper (CA1, CA2, CA3 fields) and the HDG were clearly distinguished either in the sham mice (Figure S1 a, b, e, f) or injured mice (Figure S1 c, d, g, h). While there was an obvious tissue lesion in the cortex, the hippocampus only exhibited thinner in the granular neuron layer at the ipsilateral side using either H.E. staining (Figure S1 c, d) or Nissl staining (Figure S1 g, h). The anatomic structure of hippocampus remained largely intact after TBI caused by a moderate level of impact. We did not see any global malformation in the hippocampus either in the control or injured mice.

To determine whether there is an obvious cell loss in the hippocampal dentate gyrus, we further measured the cell density in the dentate gyrus using 4′, 6-diamidino-2-phenylindole (DAPI) staining, a fluorescence that binds strongly to DNA and stains all nuclei. The results showed that the cell density in the dentate gyrus at the impact epicenter was slightly reduced compared to the sham control. Nonetheless, the difference was not statistically significant (Figure S2). Nissl stains rough endoplasmic reticulum [32], a site for protein synthesis. Decreased Nissl staining of spared neurons may be a reflection of cell stress. These results indicate that TBI caused by a moderate level of impact may lead to cell stress but does not obviously change the architecture of the hippocampus, and the majority of the mature granular neurons in the HDG survived but stressed at this level of CCI-injury.

### Moderate TBI causes significant dendrite degeneration in the granular neurons in the hippocampal dentate gyrus

**Figure 1:**
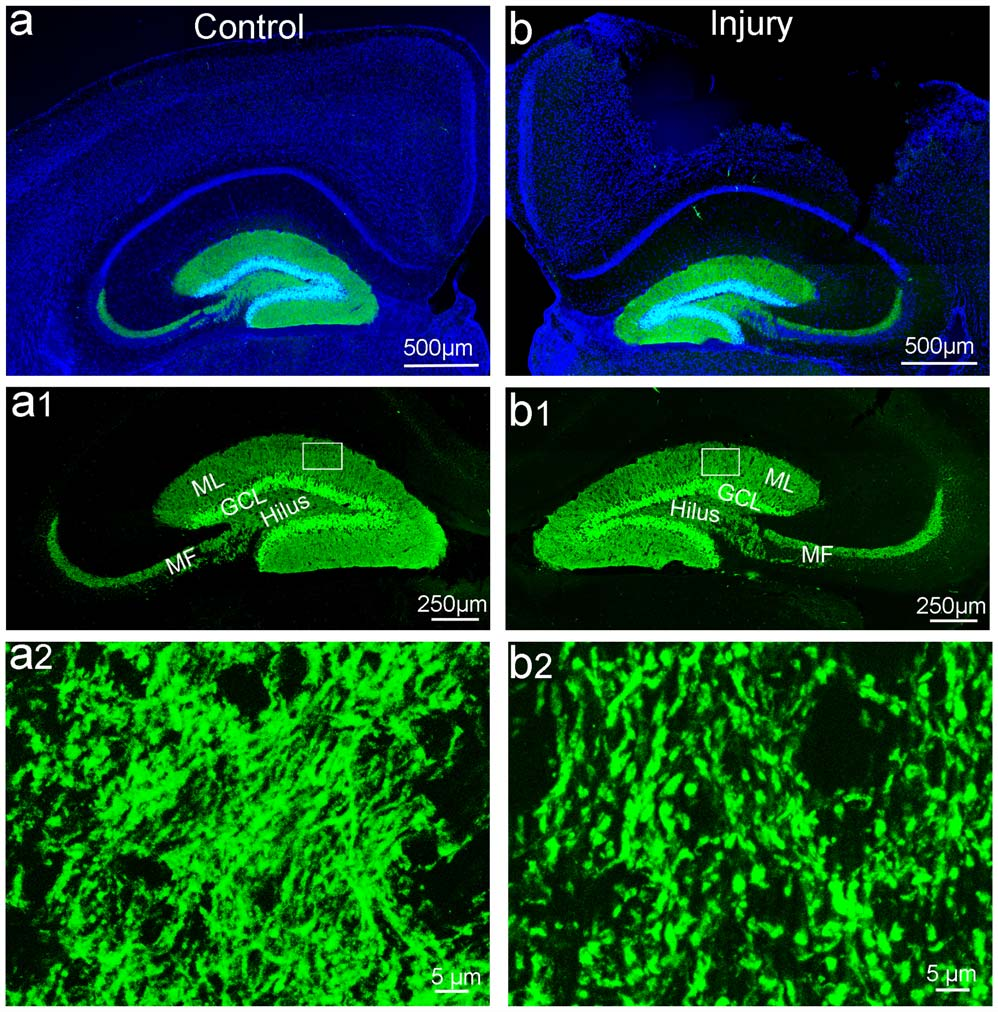
The anatomical structure of the hippocampus in POMC-Cre;Z/EG double transgenic mice. There was no gross morphological change in the HDG between moderate TBI (a, a1) and sham control mice (b, b1). EGFP staining (green) showed that there were many more disconnected bulbs and swellings in the injured HDG (b2) compared with the sham (a2).

Although most of the mature granular neurons in the HDG are spared from death following TBI, that does not mean that they are not injured or that their functions are normal. There is still a lack of data to show whether there are other injuries to these spared neurons. In order to further assess the subtle degeneration of those spared mature granular neurons in the HDG following TBI, we evaluated their dendrites, dendritic spines, and synapses following moderate cortical controlled impact (CCI) injury. We first took advantage of an unique line of genetically modified mice, POMC-Cre;Z/EG double transgenic mice, in which EGFP is so highly expressed in the DG granular neurons as to permit high resolution of their cytoarchitecture including cell bodies, dendrites, and their axonal projections (Figure 1).

The male double transgenic mice were subjected to moderate CCI injury (n = 5) or sham control treatment (n = 5) at the age of 8 weeks old as described before [24], [25], [26]. Seventy-two hours after surgery, the moderate TBI had caused a significant brain tissue lesion in the neocortex (Figure 1 b) as seen in the wild type mice [33]. In both the control and injured POMC-Cre;Z/EG double transgenic mice, EGFP-labeled granular neurons resided in the granular cell layer with dendrites projecting into the molecular layer, and axons (mossy fiber, MF) projecting toward CA3 (figure 1, a-a1, b-b1). The anatomical structure of the hippocampus remained intact both in the control (Figure 1 a-a1) and injured mice (Figure 1, b-b1), confirming our previous notion that moderate TBI does not obviously change the architecture of the HDG.

Nevertheless, at the higher magnification at which it is possible to distinguish single dendritic fibers, we found that the dendrites of granular neurons in the hippocampus dentate gyrus of sham mice were smooth, with only occasional spots of swelling in the dendrites (figure 1, a2). In contrast, we found the dendrites of granular neurons of TBI injured mice were not smooth, exhibiting disconnected bulbs and swellings (figure 1, b2). Moderate TBI significantly increased the number of swellings and disconnected bulbs from 611.2±73.5/mm^2^ in a sham control mouse to 3,944.4±246.9/mm^2^ in the injured HDG (n = 5, p<0.01). Swellings and disconnected bulbs are hallmarks of dendritic injury [17], [18], [19]. These results demonstrate that although most of the dentate gyrus granular neurons are spared following moderate TBI, their dendrites are significantly injured.

Due to the extremely high density of dendrites in the molecular layer in the dentate gyrus, the swellings and disconnected bulbs observed above on the injured dendrites were based on multiple spared granular neurons. This still does not reveal how much damage the dendrites from individual granular neurons have sustained. To further examine dendrite degeneration following moderate TBI in individual granular neurons, we assessed the morphologies of the spared neurons by Golgi staining.

**Figure 2:**
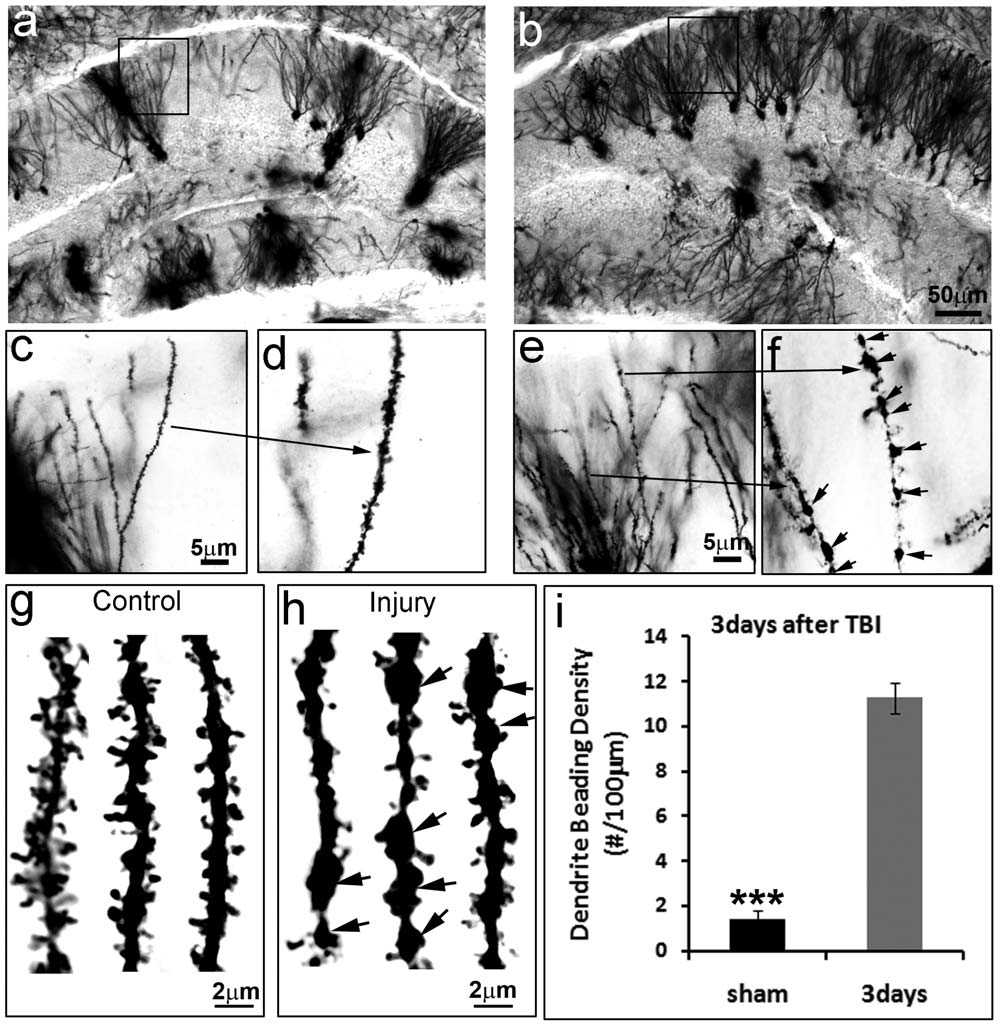
Moderate TBI causes dendrite degeneration in the HDG. Golgi staining revealed the individual neurons including their processes and spines both in the control (a, c, d and g) and the moderate CCI injured hippocampus (b, e, f and h). The dendrites and spines of the spared neurons in the injured HDG were significantly damaged. Quantitative data showed that the number of dendrite beadings, the hallmark of an injured dendrite was increased dramatically in the HDG of TBI mice HDG as compared with sham control (i). (n = 5, ***, p<0.005).

Golgi staining is an extremely reliable and sensitive method for revealing morphological details of individual neurons, especially dendritic spines [34], [35], [36]. Golgi staining made individual granular neurons and their processes visible in the control brain (Figure 2 a) and the injured brain (Figure 2 b). Higher resolution images taken of the control hippocampus corresponding to the position of the epicenter of the injured mice showed the dendrites of granular neurons with tiny protrusions of bulbous heads at this resolution (Figure 2 c and d). These protrusions of bulbous heads are dendritic spines, which will be quantified next. The dendrites extending to the molecular layer in the control mice were smooth with very few beadings (1.43±0.41 beading/100 µm length of dendrite) (Figure 2 c, d, g and i). In contrast, the dendrites of the spared granular neurons in the injured dentate gyrus were dramatically swollen with beadings (Figure 2 e and f, pointed out by arrows). The number of beadings increased to 11.27±0.83 beading/100 µm of dendrite (Figure 2 h and i). Furthermore, the density of the dendritic spine was dramatically reduced, which will be quantified later.

**Figure 3:**
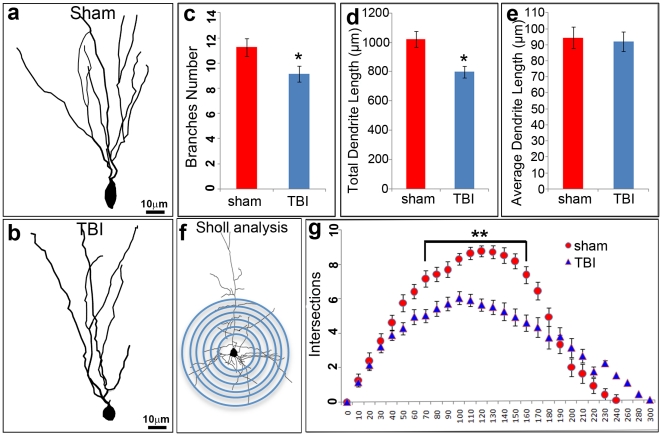
Moderate TBI attenuate dendrite complexity in the HDG. Neurolucida reconstruction of Golgi stained granule neurons in the HDG from sham mice (a) and moderate TBI mice (b). Both the dendrite total length and branches were decreased in spared neurons in HDG after TBI (c, d). There was no significant difference in average dendrite length between the 2 groups (e). Sholl analysis-derived distribution of granule neuron dendritic complexity and mean number of intersections of dendrite branches with consecutive 10 µm-spaced concentric spheres (f). A dramatic reduction of dendritic complexity was found in spared neurons in TBI mice compared with sham control. (n = 5, *, p<0.05, **, p<0.01).

We reconstructed dendritic morphologies of individual granular neurons (n = 100) in the HDG from control mice (figure 3 a) and injured mice (figure 3 b) and assessed whether their complexity had been reduced by the damage. The average number of dendritic branches decreased from 11.27±0.64 to 9.13±0.91 after moderate TBI (Figure 3 c) (p<0.05). The average of total dendrite length decreased from 1019.95±39.92 µm to 798.01±54.7 µm (Figure 3 d)(p<0.05), while the average length of each branch was slightly shorter, but not statistically different (Figure 3 e) (p>0.05).

We then performed Sholl analysis (Figure 3 f), a quantitative method for morphometric neuronal studies, to better understand the dendritic branching characteristics of individual neurons in the injury area and the corresponding control regions. Scholl analysis revealed that spared granular neurons showed reduced dendritic complexity compared to the sham controls (3 g). This decreased complexity was particularly evident at a distance between 70 and 170 µm from the soma (Figure 3 g). These results confirm that although most of the mature granular neurons in the HDG are spared following moderate TBI, their dendrites are extensively injured. Thus the actual injury in the hippocampus is much more severe than assumed before using cell death as an indicator.

### Degeneration of dendritic spines in the HDG granular neurons following moderate TBI

**Figure 4:**
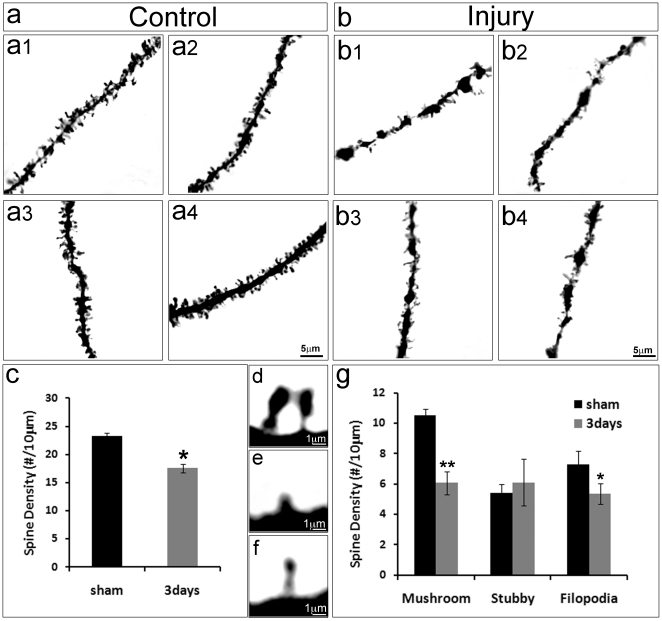
Moderate TBI leads to spine degeneration in the HDG. High power images of a single spine in control mice (a, a1–a4) and moderate TBI mice (b, b1–b4). Three types of spine, including mushroom (d), stubby (e) and fliopodia (f). Quantitative data showed that the density of the dendritic spine was significantly reduced in the spared neurons in the injured HDG (c), especially in mushroom and filopodia shaped spines (g). (n = 5, *, p<0.05, **, p<0.01).

We next investigated whether CCI injury and dendritic damage leads to spine degeneration. We imaged the dendritic spines in the granular neurons of control (Figure 4 a) and moderate TBI-injured mice (Figure 4 b) and determined their density and characteristics. To avoid variations in spine density along the dendrite, spines located at similar positions, 5–30 µm from the tip of each dendritic branch were selected for comparison. The results showed that the density of the dendritic spines was significantly reduced in the spared granular neurons in the injured mice from 23.27±0.61 to 17.55±1.38 per 10 µm length of dendrite (Figure 4 c) (p<0.05). This indicates a reduction in the number of dendritic spines in the spared neurons following moderate TBI.

Furthermore, we also found that the sizes of dendritic protrusions were obviously smaller in the injured granular neurons (Figure 4 a and b). The protrusion of dendritic spines was categorized into 3 classes based on their shapes and lengths: mushroom-shaped spines, shaped so that the diameter of the head is much larger than the diameter of the neck (Figure 4 d) and usually seen in mature synapses [37]; stubby-shaped spines (Figure 4 e), short and wide in shape and considered to represent either a transitional growth stage between an early to mature spine or the stage during retraction of the mature spine for elimination; and filopodia-shaped spines, (Figure 4 f), which are highly mobile, long and thin with a fine tip, and seen at the early stage of spine formation [38], [39], [40], [41]. In order to visualize each spine at high resolution, images with z-stack were taken, and reconstructed to show the morphologies of each spine.

When we analyzed the density of dendritic spines for each type, we found that the density of mushroom-shaped spines decreased dramatically (10.55±0.39/10 µm in the control granular neurons, 6.68±0.05/10 µm in the injured granular neurons, p<0.01); the filopodia-shaped spines showed significant reduction from 7.29±0.91/10 µm in the control neurons to 5.36±0.14/µm in the injured neurons (p<0.05); while the stubby-shaped spines slightly increased (5.43±0.54/10 µm in the control neurons, 6.11±0.98/10 µm in the injured neurons), but did not show any significant differences (figure 4 g). These results indicate that moderate TBI not only damages the spared neurons, but the number of spines on the injured dendrites is also reduced. It also suggests that the density of functional synapses is reduced, and the capacity to re-form new synapses is impaired in the spared neurons following moderate TBI.

### Synaptic density was reduced in the hippocampus following moderate TBI

**Figure 5:**
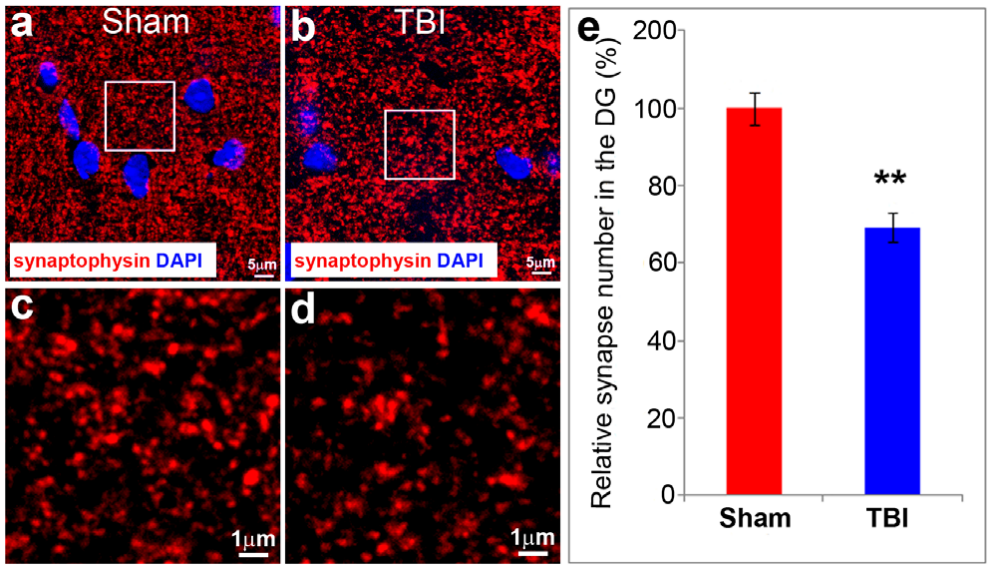
Moderate TBI decreases the number of synapses in the injured area. Synaptophysin staining (red) reveals the pre-synaptic puncta in the molecular layer of the HDG in control (a, c) and moderate TBI mice (b, d). Quantitative data showed a significant decrease of synapses in the HDG of injured mice (e). (n = 5, **, p<0.01).

We performed immunostaining with an antibody against synaptophysin, a synaptic vesicle glycoprotein, to determine if the reduced density of dendrite and dendritic spines in the hippocampal granular neurons of TBI injured mice leads to a reduction in synapse density. Its ubiquity at the synapse has led to the use of synaptophysin immunostaining as a marker for quantifying synapses [42]. Synaptophysin exhibited punctate patterns in both controls (figure 5 a) and TBI (figure 5 b). The results showed that synaptophysin immunostaining occurred in a punctate distribution consistent with localization of presynaptic boutons. In contrast, the cell nuclei showed negligible synaptophysin immunoreactivity (Figure 5 a, b). We then measured synapse density based on synaptophysin immunostained puncta in the molecular layer of the HDG.

We quantified the density of synaptophysin-positive punctates in the molecular layer of the hippocampus in the epicenter of the TBI-injured mice (Figure 5 b, d) or at the same anatomical position in the brain of control mice (Figure 5 a, c). The result showed that in the TBI-injured brain the density of the synaptophysin-positive punctates in the epicenter was reduced to 69.23% compared to the corresponding region in sham control (p<0.01). This result indicates that moderate TBI decreases the number of synapses in the HDG.

### Decrease of intrinsic membrane excitability of granule cells in the hippocampus after TBI

**Figure 6:**
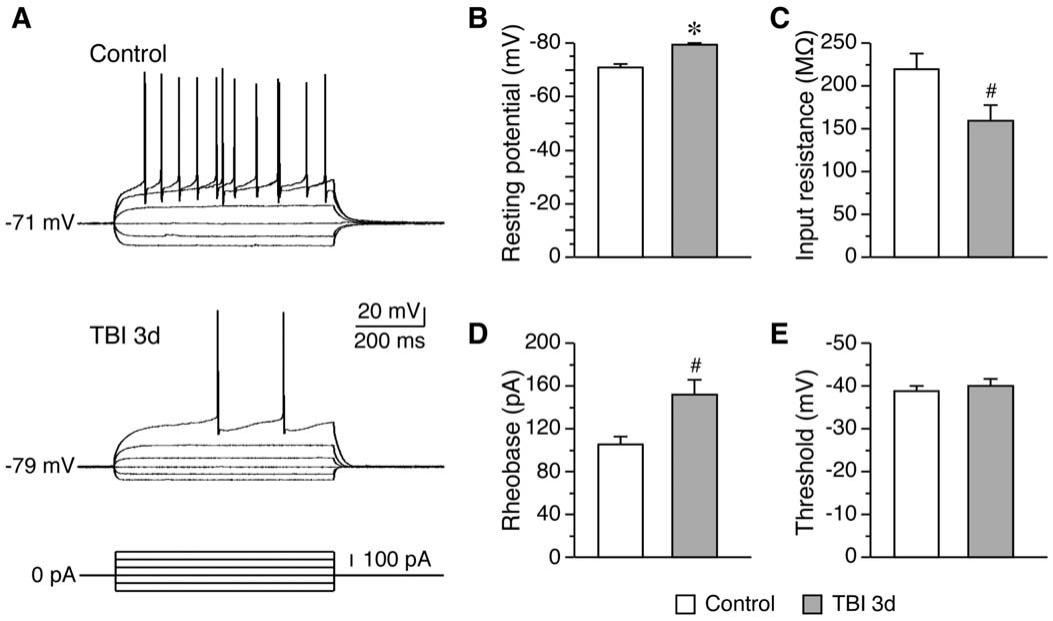
Decreased neuronal excitability of granule cell after TBI. (A), representative traces showing the voltage responses of granule cells to current pulses. The same amount of positive current evoked fewer spikes in post-traumatic cells. Meanwhile, post-traumatic cells exhibited smaller responses to negative currents. (B–E), summary data showing the post-traumatic changes of resting membrane potential (B), input resistance (C), and rheobase (D) in granule cells. The spike threshold showed no change after TBI (E). *, *P*<0.01; #, *P*<0.05.

To assess whether dendritic degeneration and synaptic elimination of granular neurons led to their activity deficits, we examined the effects of TBI on the intrinsic membrane excitability of granule cells. As shown in figure 6, the resting membrane potentials were significantly hyperpolarized after TBI (control: −70.8±1.3 mV, n = 8; TBI 3d: −79.3±0.8 mV, n = 6; *P*<0.01). In addition, the input resistance in post-traumatic cells was decreased (control: 219.2±18.2 MΩ, n = 8; TBI 3d: 159.2±18.0 MΩ, n = 6; *P*<0.05). Accordingly, the rheobase, a minimal current amount required to evoke the first spike, was increased in post-traumatic cells (control: 105.0±8.0 pA, n = 8; TBI 3d: 151.7±14.5 pA, n = 6; *P*<0.05). However, the spike threshold showed no significant change after TBI (control: −38.8±1.2 mV, n = 8; TBI 3d: −40.0±1.7 mV, n = 6; *P*>0.05). These data indicate that TBI causes a long-lasting depression of neuronal excitability in granule cells.

### Inhibition of excitatory synaptic activity in granule cells after TBI

**Figure 7:**
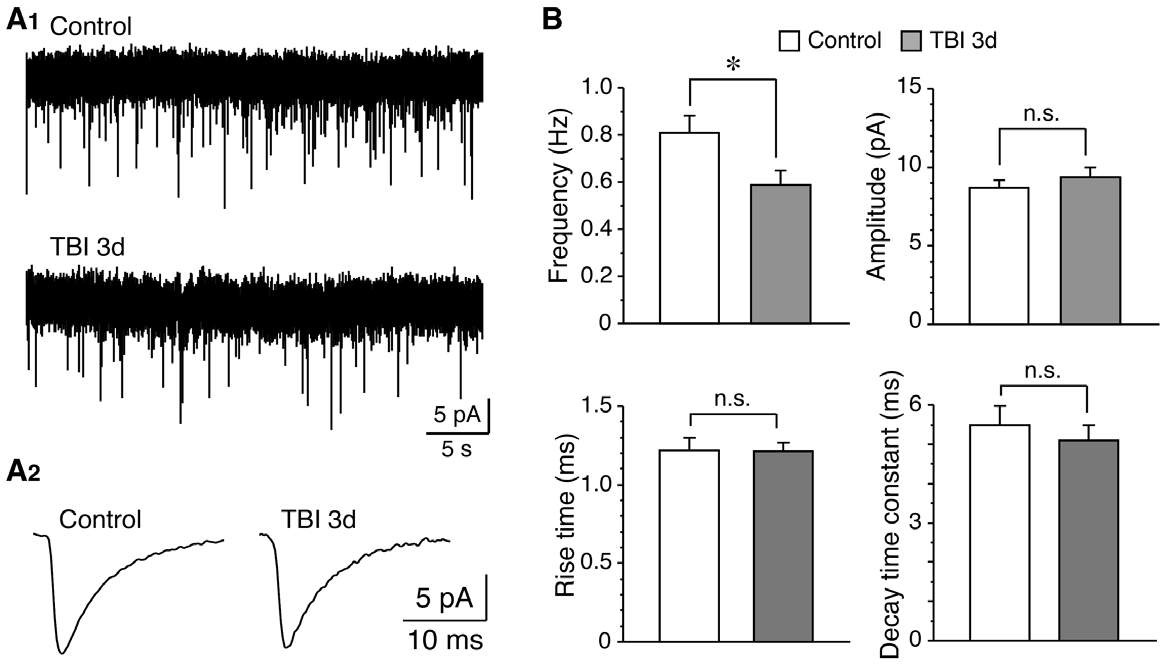
Transient inhibition of excitatory synaptic inputs in granule cell after TBI. (A), representative traces of mEPSCs recorded from control and post-traumatic cells, showing at lower scale (A1) and larger scale (A2), respectively. The traces in A2 were averaged from 150–260 events. (B), summary data showing the decrease of mEPSCs frequency, but not amplitude, rising time, and decay time constant. *, *P*<0.01.

To test whether excitatory synaptic inputs in granule cells were altered after TBI, mEPSCs were compared between control and post-traumatic cells. As shown in figure 7, we found that the frequency of mEPSCs was significantly decreased 3 days after TBI (control: 0.81±0.07 Hz, n = 5; TBI 3d: 0.59±0.06 Hz, n = 6; *P*<0.05). However, no obvious change was detected in the amplitude of mEPSCs (control: 8.74±0.49 pA, n = 5; TBI 3d: 9.40±0.63 pA, n = 6; *P*>0.05). In addition, the kinetics of mEPSCs were not altered after TBI. The rising time of mEPSCs was 1.22±0.08 ms (n = 5) and 1.21±0.06 ms (n = 6) in control and posttraumatic neurons, respectively, and the decay time constant was 5.50±0.48 ms (n = 5) and 5.09±0.41 ms (n = 6) in control and posttraumatic neurons, respectively. These findings demonstrate a decrease of excitatory synaptic inputs in granule cells after TBI.

## Discussion

Hippocampal-associated learning and memory impairments were previously thought to result from rapid cell death [43], [44], [45], [46]. Here we showed that most of the mature granular neurons in the HDG were spared following TBI at a moderate level of impact. Despite their survival, these neurons exhibited dramatic dendrite damage, dendritic spine degeneration, synapse loss, and activity impairment, contributing to hippocampal-associated learning and memory impairments following TBI. These data support the notion that dendrite degeneration and synapse elimination may significantly contribute to neurological disorders following TBI. This notion might partially explain the rapid and dramatic impairment of learning and memory during the acute phase.

The loss of neurons in the hippocampus contributes to the impairment of learning and memory following TBI. Thus, impairment of cognitive function has long been thought to be the result of rapid cell death following TBI in humans [1], [44], [45]. The distribution and amount of cell death in the hippocampus varies by injury model [47], [48], [49], injury severity [2], [50], [51], and age differences [52]. Nonetheless, the amount of neuronal death in the hippocampus generally does not match well with the degree of impaired learning and memory following TBI. For example, in TBI caused by a moderate level of impact with a CCI model, the injured mice show significant learning and memory impairment [53]. However, the cell death in the hippocampus is fairly mild, and most of the mature granular neurons in the hippocampus are spared following moderate TBI [2], [5], [54], [55]. Further study shows that the dead cells in the hippocampus are predominately immature granular neurons [25], [55], while the majority of mature granular neurons in the dentate gyrus are spared in CCI injury model. Mild TBI can also cause prolonged spatial memory deficits without showing either neuronal cell death or axonal injury in hippocampus [56]. These data suggest that there are other factors, besides those dying neurons in the cortex and hippocampus including HDG, CA1 CA3 and hilus that contribute to the rapid impairment of learning and memory in the acute and subacute phases following moderate CCI injury.

Previous studies have shown that injured animals demonstrated a diffusive axonal injury [10], [11], [12], which could contribute to functional impairments. On the other hand, dendritic arbors may function as antennas that detect specific sensory stimuli or as domains for receiving or providing synaptic inputs or outputs to target cells. Maintaining such complex dendrite arbors is a prerequisite for synapse formation, neuronal connections, and to a large degree, the function of the neuron [57]. We determined the morphologies of the spared neurons by using Golgi staining, an extremely reliable and sensitive technique for demonstrating morphological details in individual neurons [34], [58]. Golgi staining revealed significant degeneration of the granular neurons in the injured HDG. The dendrites showed swelling with beading, a hallmark of dendritic injury [17], [18], [19] (Figure 1, 2 and 4).

Dendritic arbors are a vital component of neurons that play a critically important role in providing a massive receptive area on the neuronal surface for spine formation. As many as 30,000∼40,000 spines are present on each large pyramidal neuron. Thus damage in dendrites may result in significant decreases in the number of dendritic spines and synapses. This decrease could significantly contribute to a decline in hippocampal-associated learning and memory exhibited in TBI patients following injury. Our unpublished data showed that the degree of dendrite degeneration in the hippocampus reduced over time after the injury, suggesting plasticity in the dendrites of the adult brain. Despite this plasticity, dendrite degeneration post injury could also be long-lasting.

Another study found that moderate to severe levels of traumatic brain injury with fluid percussion model still showed significant shortening of layer V/VI basal dendrite arbors 4 months post-injury [59]. The molecular mechanisms that regulate dendritic damage are poorly understood. It would be interesting to investigate whether dendrite damage is one of the events induced by excitotoxicity, or if it is a secondary effect of axonal injury [60]. It would also be beneficially to investigate methods to increase the plasticity of dendrites after injury.

Spines constitute the regions of dendritic arborization that receive most of the excitation input [61]. The function of the nervous system relies on the establishment and maintenance of synaptic connections between the presynaptic and postsynaptic spines from neurons and their specific target cells. Further analysis showed that compared to the control, the density of dendritic spines was significantly decreased in the injured neurons in the ipsilateral hippocampus (Figure 4). Protrusions of spines vary in shape and length, and are generally separated into three classes: filopodia, stubby, and mushroom-shaped spines. Mushroom-shaped spines are large in size and are the postsynaptic spines of mature synapses, whereas the filopodia- and stubby- shaped spines are small in size and are in the process of forming new synapses [37], [38], [39], [40]. Each type of spine represents different stages during synaptogenesis.

We further assessed each type of spine in the dentate gyrus granular neurons following TBI. Analysis of the structural properties of spines (figure 4) showed that the number of mushroom-shaped and filopodia-shaped spines was significantly decreased in the ipsilateral side. There was no significant difference in the stubby-shaped spines. The mushroom-shaped spines were mature spines forming synapses. A reduction in the density of the mushroom-shaped spines suggests a reduction in the number of the synapses, which was confirmed by synaptophysin staining (Figure 6). A reduction in the density of the filopodia-shaped spines suggests an impairment in spine reformation at this time point after injury. These data collectively indicate a very extensive injury to the dendrites and spines of the spared neurons in the injured area.

Synaptic loss in the adult hippocampus correlates directly with the decline in cognitive performance seen in epilepsy [62], aging [63], [64], [65], and neurodegenerative diseases [66], [67]. Our electrophysiological recordings indicated an inhibition of background excitatory inputs in dentate granule cells early (3 days) after TBI. The cellular mechanisms underlying the changes in excitatory inputs are unclear. Granule cells receive extensive excitatory inputs from mossy cells in the hilus. Numerous studies have shown that mossy cells are highly vulnerable to TBI [1], [68], [69]. Therefore, the post-traumatic loss of mossy cells may contribute to the depression of mEPSCs. In addition, the degeneration of dendritic spines and loss of functional synapses may also result in a reduction of excitatory synaptic activity. This possibility is also supported by our observation that TBI produced a decrease of mEPSC frequency, but not mEPSC amplitude. On the other hand, a depression of membrane excitability was observed in granule cells following the injury. Dentate granule cells express inwardly rectifying potassium channels [70]. An increased activity of these channels has been observed in granule cells in temporal lobe epilepsy, leading to a decrease of input resistance [71]. A similar mechanism might underlie the posttraumatic depression of neuronal excitability in dentate granule cells, since inwardly rectifying potassium channels are very sensitive to TBI [72]. The results from the present study provide a potential anatomic substrate and electrophysiological activity to explain, in part, the development of posttraumatic memory deficits. They also indicate that neurodegeneration in the HDG may serve as a new therapeutic target following TBI. Meanwhile the membrane excitability may be dynamic due to the plasticity of dendrites, spines and synaptic activity following injury. Indeed, when recording the electrophysiological activity weeks after injury, the granular neurons exhibit higher membrane excitability, which may contribute to the etiology of epilepsy in the chronic phase following TBI [68], [73].

### Conclusion

This study shows that TBI not only causes neuron death, but also induces dramatic dendritic and spine degeneration, leading to reduction in the number of synapses. Furthermore, synapse loss can also be seen in aging and pathological conditions such as Alzheimer's disease [74]. Currently, how a synapse is eliminated in pathological conditions is still a mystery, and there is no effective treatment to prevent synapse loss. Neurodegeneration of spared neurons following TBI hasn't been adequately studied and may serve as an important therapeutic target to increase functional recovery. TBI-induced synaptic elimination might provide a unique model to study synaptic elimination in the adult brain. Understanding the molecular and cellular mechanisms that regulate synaptic loss following TBI will advance the understanding of the pathophysiology following TBI. This may also suggest an innovative strategy for neuroprotective treatments to improve post-traumatic neurological recovery, and shed light on the development of therapeutic treatment for other neurodegenerative diseases.

## Supporting Information

Figure S1 **Histological manifestations of moderate TBI in mice.** At 3 days post-TBI, the brains' gross pathology examination showed no significant morphological change in the hippocampus of moderate TBI mice compared with sham control. H&E (a–d) and Nissl (e–h) staining showed that tissue of the hippocampus in the injured brain was intact without any dramatic malformation.
(TIF)

Figure S2 **Cell lost in Hippocampus of moderate TBI mice.** DAPI (blue) staining revealed the nuclei of all cells at the injury site of the hippocampus (a, c and e sham control, b, d and f moderate TBI). Counting results show that the total cell density based on the DAPI staining was slightly reduced in HDG at the epicenter compared to the sham control (g) (n = 5, p>0.05).
(TIF)
