# Supplementary figures and images for: Moderate Traumatic Brain Injury Causes Acute Dendritic and Synaptic Degeneration in the Hippocampal Dentate Gyrus
Source: PLoS One. 2011 Sep 13;6(9):e24566. doi: 10.1371/journal.pone.0024566 (PMC3172233; doi:10.1371/journal.pone.0024566)

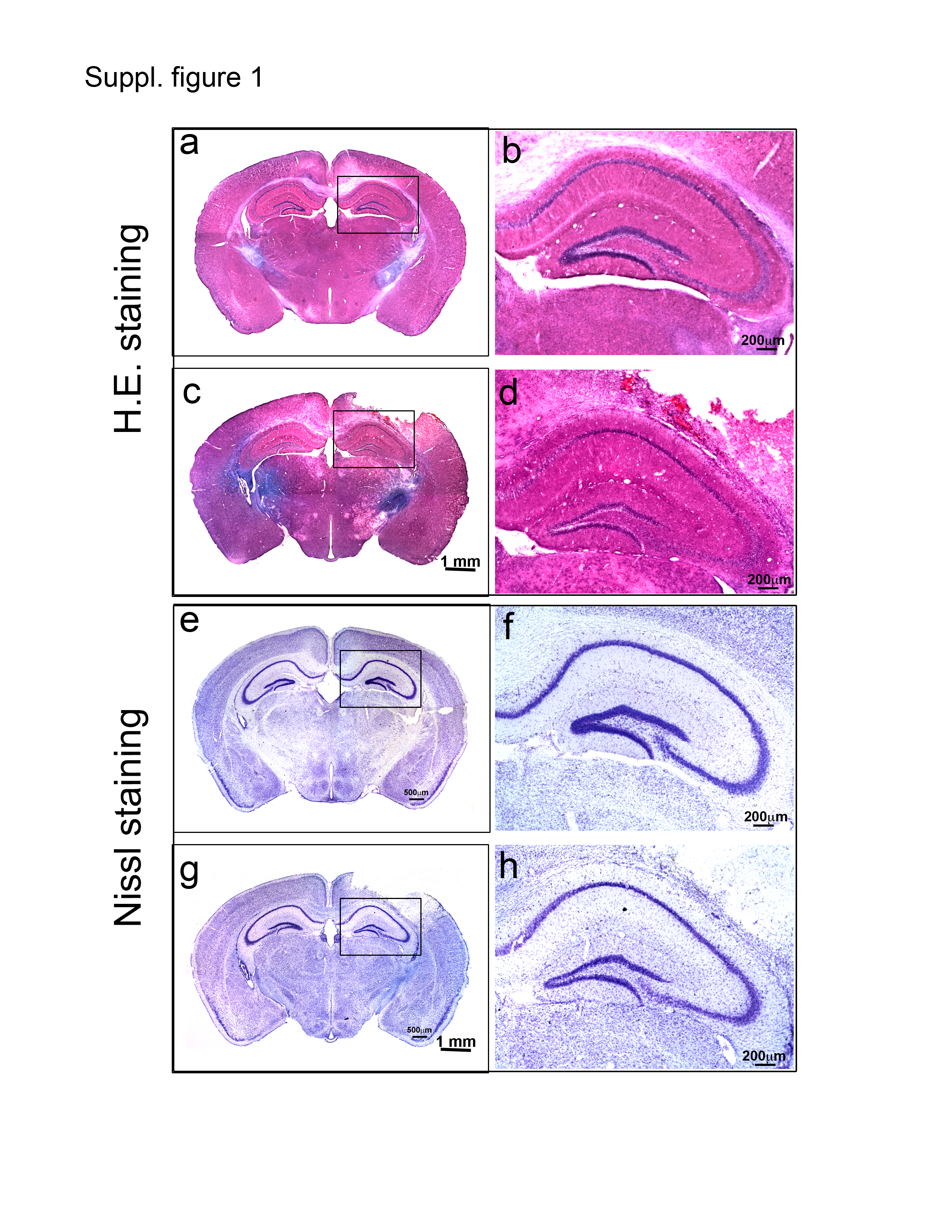

Supplement: Figure S1 — Histological manifestations of moderate TBI in mice. At 3 days post-TBI, the brains' gross pathology examination showed no significant morphological change in the hippocampus of moderate TBI mice compared with sham control. H&E (a–d) and Nissl (e–h) staining showed that tissue of the hippocampus in the injured brain was intact without any dramatic malformation. (TIF) [file pone.0024566.s001.tif]

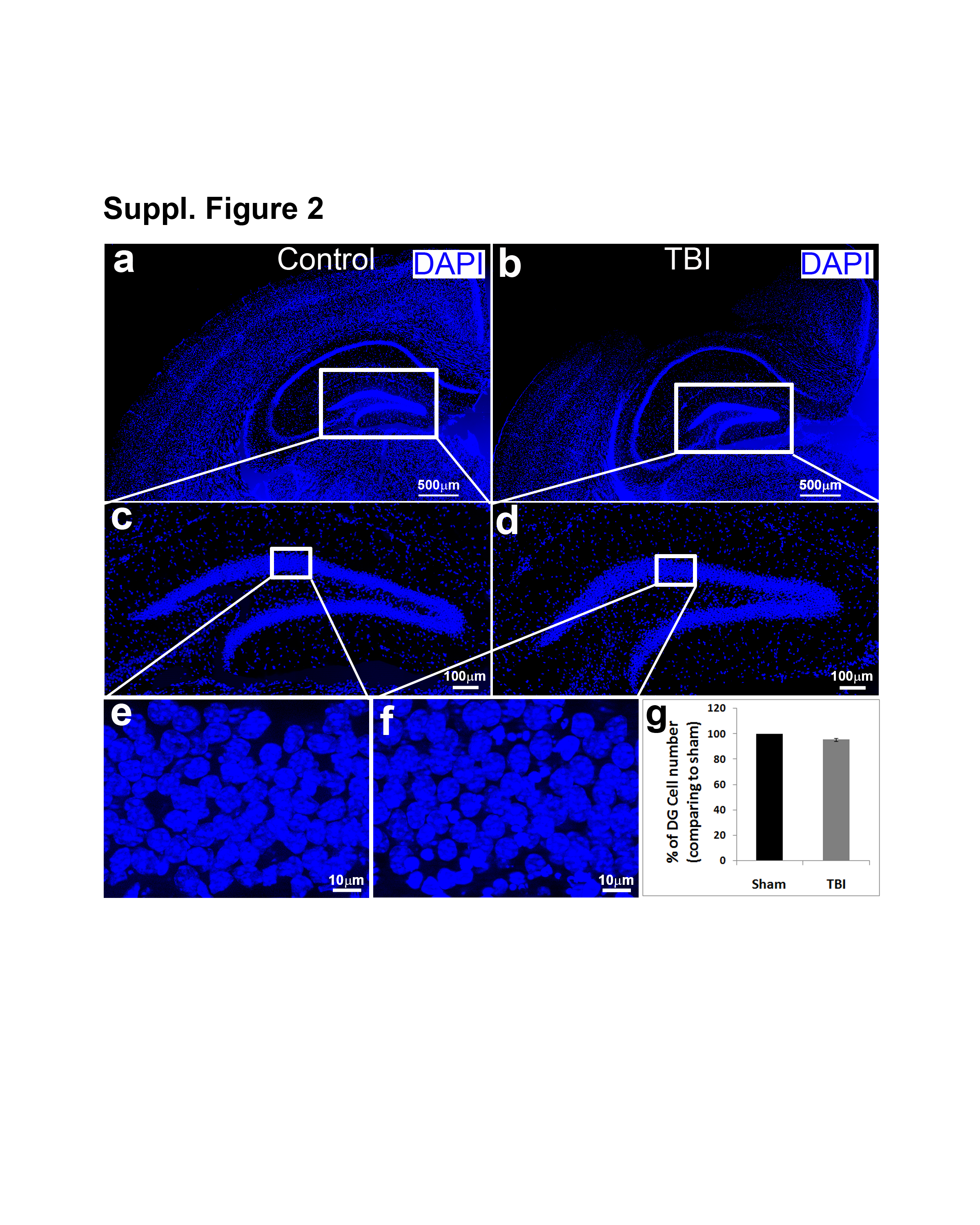

Supplement: Figure S2 — Cell lost in Hippocampus of moderate TBI mice. DAPI (blue) staining revealed the nuclei of all cells at the injury site of the hippocampus (a, c and e sham control, b, d and f moderate TBI). Counting results show that the total cell density based on the DAPI staining was slightly reduced in HDG at the epicenter compared to the sham control (g) (n = 5, p>0.05). (TIF) [file pone.0024566.s002.tif]
